# Progress in Diseases of the Heart

**Published:** 1902-02-01

**Authors:** 


### Progress in Diseases of the Heart.

**Tuberculosis of the Pericardium.**—Riesman[1] has recently published the results of a careful study of tuberculosis of the pericardium. He says it may (1) be a part of a general miliary tuberculosis; or (2) associated with general serous membrane tuberculosis, though not commonly, for more often it is the pleura and the peritoneum which are involved together, the pericardium escaping; (3) the majority of cases of well-marked pericardial tuberculosis are the result of extension from neighbouring tuberculous foci—most frequently from the mediastinal and peribronchial lymph glands. Ordinary pulmonary tuberculosis does not often lead to pericarditis. In the presence of well-marked tuberculosis of any of the neighbouring organs, an associated pericarditis may be suspected, particularly if obliterative, to be tuberculous; but in the absence of well-marked tuberculous disease elsewhere there is frequently nothing to call attention to the possibility of such an origin. Tuberculous pericarditis may occur at any time of life, in children and in adults, but most commonly in men. Its symptoms are usually those of adherent pericardium, though they may be those of effusive pericarditis. It is the former which is most difficult of diagnosis. That the condition is tuberculous is more a matter of subsequent reasoning than of actual demonstration by physical signs. In Riesman's own case the manifestations were cardiac failure, as shown by dropsy, cyanosis, and dyspnœa on exertion; the presence of a friction sound early in the disease; the absence of signs of effusion and of endocardial murmurs; the imperceptibility of the apex beat, and the persistently feeble pulse. All these signs pointed strongly to adherent pericardium. This condition, however, may give rise to other physical signs. Thus there may be an absence of impulse without any augmentation in the area of precordial dulness. In certain cases where mediastinitis has caused adhesion of the heart to the chest wall in front there may be a marked systolic retraction of some of the ribs on the lateral or posterior aspect of the chest, and this may be immediately followed by a shock, which can be felt by the hand, due to the elastic recoil or rebound of the chest wall at the beginning of diastole as soon as the systolic dragging force has ceased. A complete arrest of the respiratory movements in the epigastric triangle is also a common sign, though not pathognomonic. Another symptom which deserves mention is the early appearance of ascites before the onset of œdema of the lower extremities. The diagnosis of tuberculosis, that of adherent pericardium being made, depends on the exclusion of the ordinary causes of adherent pericardium, such as rheumatism and other acute infections. The presence of a hæmorrhagic effusion in the pleural cavity would be of signal service in diagnosis. The main points of distinction from a rheumatic affection are that the size of the heart is usually normal, endocardial murmurs are absent, and there is no history of rheumatism. The duration of the disease is usually from four to eight months. The medical treatment is entirely palliative and expectant.

**Gelatin.**—Gebele [2] reports two cases of epistaxis and one of bleeding from a tooth socket in a hæmophiliac, which could not be controlled by ordinary means and were successfully treated by the use of gelatin injections. He also made experiments on dogs to determine the conditions of success. A considerable loss of blood favours the absorption of the gelatin solution which increases the coagulability of the blood; whereas if the hæmorrhage has been but small the action of the gelatin will be inadequate. A 2 per cent. strength for subcutaneous and a 10 per cent. strength for local use are very serviceable. Barth [3] describes a case of aneurysm of the ascending part of the aortic arch treated with gelatin injections. The signs and symptoms at the time of treatment were slight cyanosis of the lips and cheeks, enlarged veins in the upper thoracic wall, radial pulses soft and the right one a little smaller and later than the left; a swelling in the right intra-clavicular region was well marked and showed pulsations. The dulness extended equally in all directions and measured $4\frac{1}{3}$ inches in diameter. There was a loud systolic murmur all over the cardiac area and over the swelling. Two courses of injection were carried out, each consisted of 12 injections of a 1 per cent. solution of gelatin administered on alternate days. Examination after the last course showed that the swelling felt firmer, was not so prominent, nor did it pulsate as visibly, and the area of dulness had shrunk to an area of $2\frac{1}{3}$ inches by 2 inches. The subjective symptoms—of pain, discomfort and inability to make any exertion—had completely disappeared. In a recent discussion [4] on the diagnosis and treatment of aortic aneurism at the Medical Society of London, two speakers described unfavourable results from the injection of gelatin solutions—in one case a fatal issue with symptoms of cerebral thrombosis, comparing closely with cases described in Germany. A third, however, claimed good results—evidence of consolidation—in four cases, though one of the four contracted fatal tetanus, or what was believed to be tetanus, though the most stringent antiseptic precautions had been instituted. Probably the presence of tetanus bacilli or spores was an accidental occurrence, for it is difficult to believe that the spores could have been in the original gelatin, and resisted the prolonged and thorough sterilising processes to which it was subjected.

**A Tracheal Tug** was, when first discovered, thought to be pathognomic of aneurysm of the transverse arch of the aorto. It was subsequently found to be present in many normal persons, and also in some cases of tumours within the chest. Sewall's [5] attention was attracted to the phenomenon by finding no aneurysm in a patient who, during life, had had a distinct tracheal tug. There was, however, adhesion of the left pleura to the chest wall, and induration of the root of the left lung. Now it is clear that the downward stroke of the transverse aorta on the left bronchus is expended partly on the trachea, partly on the upper lobe of the left lung, and normally stretches the latter rather than pulls down the relatively fixed trachea. But if the left lung is fixed to the chest wall by adhesions, and especially if there is any fibrosis, it cannot be pulled down or stretched, and there is a tracheal tug. Examination of some hundreds of individuals confirmed this—a tracheal tug quite palpable in character is, in the majority of cases, associated with and dependent upon adhesions of the left pleura. Diminished extensibility of the lung tends to produce the same phenomenon, and the tug is most pronounced when the conditions are combined. The tug varied from a just perceptible impulse transmitted to the observer's finger at the end of inspiration to a strong downward twitch of the larynx approaching in vigour that occurring in cases of aneurysm.

1 Amer. Jour. Med. Science, July 1901. 2 Med. Re-., July 6, 1901. 3 Münch. Med. Woch., April 2, 1901. 4 Brit. Med. Jour., Sept. 7, 1901. 5 Amer. Jour. Med. Science, Aug. 1901.

